# A Framework for Understanding the Emerging Role of Corticolimbic-Ventral Striatal Networks in OCD-Associated Repetitive Behaviors

**DOI:** 10.3389/fnsys.2015.00171

**Published:** 2015-12-17

**Authors:** Jesse Wood, Susanne E. Ahmari

**Affiliations:** ^1^Translational Neuroscience Program, Department of Psychiatry, University of PittsburghPittsburgh, PA, USA; ^2^Center for Neuroscience, University of PittsburghPittsburgh, PA, USA; ^3^Center for the Neural Basis of Cognition, University of PittsburghPittsburgh, PA, USA

**Keywords:** OCD (obsessive-compulsive disorder), compulsive behavior, amygdala, dopamine, OFC, ventral striatum, accumbens, network

## Abstract

Significant interest in the mechanistic underpinnings of obsessive-compulsive disorder (OCD) has fueled research on the neural origins of compulsive behaviors. Converging clinical and preclinical evidence suggests that abnormal repetitive behaviors are driven by dysfunction in cortico-striatal-thalamic-cortical (CSTC) circuits. These findings suggest that compulsive behaviors arise, in part, from aberrant communication between lateral orbitofrontal cortex (OFC) and dorsal striatum. An important body of work focused on the role of this network in OCD has been instrumental to progress in the field. Disease models focused primarily on these regions, however, fail to capture an important aspect of the disorder: affective dysregulation. High levels of anxiety are extremely prevalent in OCD, as is comorbidity with major depressive disorder. Furthermore, deficits in processing rewards and abnormalities in processing emotional stimuli are suggestive of aberrant encoding of affective information. Accordingly, OCD can be partially characterized as a disease in which behavioral selection is corrupted by exaggerated or dysregulated emotional states. This suggests that the networks producing OCD symptoms likely expand beyond traditional lateral OFC and dorsal striatum circuit models, and highlights the need to cast a wider net in our investigation of the circuits involved in generating and sustaining OCD symptoms. Here, we address the emerging role of medial OFC, amygdala, and ventral tegmental area projections to the ventral striatum (VS) in OCD pathophysiology. The VS receives strong innervation from these affect and reward processing regions, and is therefore poised to integrate information crucial to the generation of compulsive behaviors. Though it complements functions of dorsal striatum and lateral OFC, this corticolimbic-VS network is less commonly explored as a potential source of the pathology underlying OCD. In this review, we discuss this network’s potential role as a locus of OCD pathology and effective treatment.

## Repetitive Behaviors in Obsessive-Compulsive Disorder May Be Driven by Dysregulation of Corticolimbic and Ventral Striatal Networks

**Figure 1:**
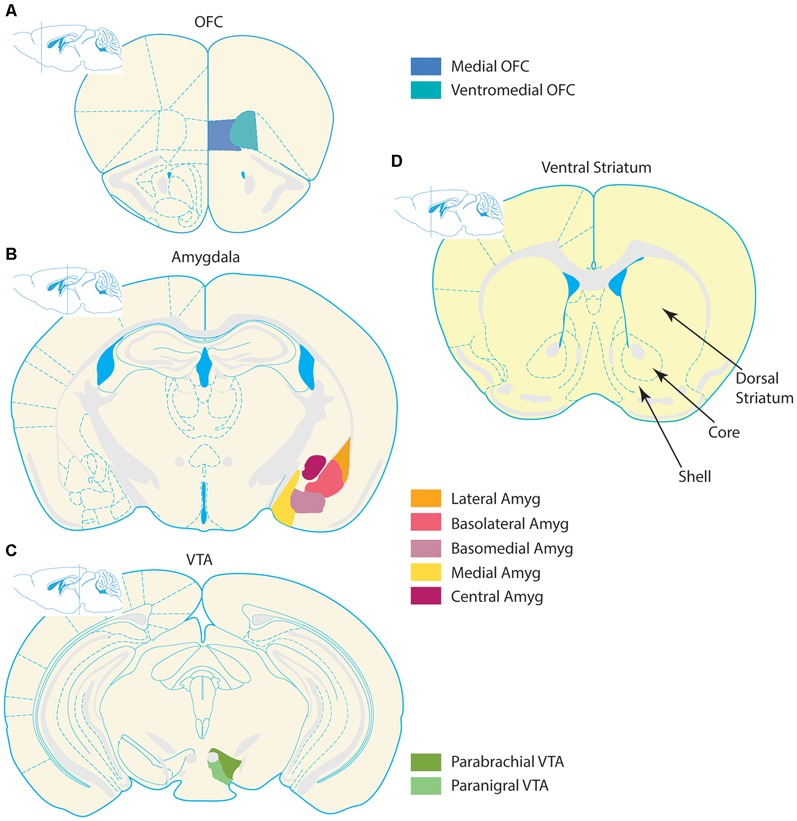
**Organization of corticolimbic-ventral striatal network.** Stylized coronal section schematics of the corticolimbic inputs to the ventral striatum (VS) are depicted alongside a schematic of the VS. Input regions are pictured in the left column, and coronal schematics are organized from top to bottom according to relative anterior to posterior locations. **(A)** Prefrontal section with the two regions comprising the medial OFC and ventromedial OFC–depicted in blue tones. **(B)** Simplified depiction of the amygdala complex in orange and violet tones. The amygdala contains several nuclei, which follow numerous naming conventions. The left hemisphere contains a more detailed depiction of subregion outlines, and the right hemisphere highlights several nuclei. The lateral, basolateral (also known as basal nucleus), and basomedial (also known as accessory basal nucleus) nuclei collectively form the basolateral complex. The cortical nucleus is not depicted in this schematic. **(C)** The two largest nuclei of the VTA, the parabrachial and paranigral nuclei, are highlighted in green tones. Some definitions of the VTA also include midline nuclei not depicted, such as the interfascicular, rostral linear, and central linear nuclei. **(D)** The VS is depicted, with special emphasis on the NAc. The core and shell regions of the NAc are denoted with arrows and bounded by dashed lines. For reference, the dorsal striatum (caudate/putamen) is notated. All schematics adapted from the 3rd edition of The Mouse Brain, by Franklin and Paxinos (1997).

The basic architecture of neuronal circuits creates a system rich with opportunities for interactions between brain regions. These interregional functional neuronal connections are fundamental to cognition, and when disrupted, can contribute to numerous pathologies (Singer and Gray, 1995; Crick and Koch, 2003; Knight, 2007; Bassett and Bullmore, 2009; Siegel et al., 2012; Moghaddam and Wood, 2014). In this review, we highlight the role of information transmission from the medial orbitofrontal cortex (OFC), amygdala, and ventral tegmental area (VTA) to the ventral striatum (VS), in producing the pathologic repetitive behaviors and dysregulated affect observed in obsessive-compulsive disorder (OCD; Figure 1). We focus on this network because: (1) the VS is an interface between striatal and limbic circuitry, and thus is uniquely poised to process affective and behavioral selection information; (2) this network is likely to be complementary to dorsal striatal networks in the context of OCD models, and is therefore an important area of study; and (3) an incomplete understanding of the neural substrates of OCD and ineffective treatments suggest a more encompassing view of the disorder is necessary. Our central goal is to illustrate that the convergence of these inputs in the VS may underlie the role of the corticolimbic-VS network in producing repetitive behavioral selection in OCD. We suggest that the simplified framework proposed here can be used to understand the circuit mechanisms underlying OCD symptomatology.

### Clinical Features of Obsessive-Compulsive Disorder

OCD affects 2–3% of the world’s population, greatly diminishes quality of life, and is a leading cause of illness-related disability (Bystritsky et al., 2001; Milad and Rauch, 2012; Subramaniam et al., 2013; Pauls et al., 2014; Ahmari and Dougherty, 2015). OCD is a heterogeneous disorder, and the obsessive thoughts and accompanying compulsive behaviors that define OCD are often conceptualized as either four or five distinct symptom dimensions, that potentially map onto distinct neuronal substrates (Mataix-Cols et al., 2005; Pinto et al., 2007; American Psychiatric Association D-TF, 2013; Pauls et al., 2014). Examples of obsession-compulsion pairs in these categories include: (1) intrusive thoughts regarding harm or doubt, and compulsive checking behavior; (2) obsessions with symmetry, and compulsive ordering, counting, and repeating; (3) taboo obsessive thoughts, and compulsive neutralizing thoughts or behaviors; (4) obsessive thoughts about contamination and compulsive cleaning and sanitation; and (5) obsessive thoughts about hoarding paired with hoarding behavior. However, it should be noted that hoarding disorder is a separate diagnostic category in the most recent version of the Diagnostic and Statistical Manual (DSM-5; Mataix-Cols et al., 2005; Pinto et al., 2007; American Psychiatric Association D-TF, 2013; Pauls et al., 2014). Although patients with OCD often have symptoms in multiple dimensions, and symptom content can fluctuate throughout the course of illness, obsessive thoughts, compulsive behaviors, and/or mental rituals are generally present in all OCD patients.

### Affective Dysregulation is a Critical Component of OCD Pathology

Though the DSM-5 classifies OCD and related disorders as a separate entity, based on potential unique neurobiological substrates, it was originally categorized as an anxiety disorder in older versions of the DSM (Stein et al., 2010; American Psychiatric Association D-TF, 2013). How to classify OCD, and its relationship to other anxiety disorders, is a topic of ongoing debate in the field (Nutt and Malizia, 2006; Craig and Fineberg, 2008; Bienvenu et al., 2012). Regardless, it is clear from both clinical presentation and ongoing research that anxiety is an important component of the disorder. Patients with OCD compulsively engage in repetitive or ritualistic behavioral sequences, which are typically performed to alleviate or avoid the severe distress and anxiety that often accompanies obsessive thoughts (Stein et al., 2010; Milad and Rauch, 2012; de Haan et al., 2013; Pauls et al., 2014; Ahmari and Dougherty, 2015). High levels of anxiety, or even panic, may occur if compulsive behaviors cannot be executed (Nutt and Malizia, 2006; Stein et al., 2010). Thus, for many patients, anxiety links the two defining features of OCD: obsessions and compulsions.

The fact that anxiety typically serves as a key moderator of OCD symptoms underscores the fact that affective dysregulation is a critical component of OCD pathology. In keeping with this idea, deep brain stimulation targeting the nucleus accumbens (NAc) core has been found to improve both obsessive-compulsive symptoms and anxiety (Mantione et al., 2015). Additionally, cessation of NAc deep brain stimulation worsens scores on the Yale-Brown Obsessive Compulsive Scale (YBOCS) and the Hamilton Anxiety Rating Scale (HAM-A; de Koning et al., 2011; Ooms et al., 2014). Improvements in both obsessive-compulsive and affective symptoms by a single treatment suggest an underlying connection between affective dysregulation and OCD, strengthening the idea that emotional dysregulation is critical to promoting OCD symptoms. This is supported by epidemiological evidence showing that anxiety disorders, particularly social anxiety, panic disorder, and generalized anxiety disorder, have higher than chance levels of comorbidity in patients with OCD (Black et al., 1992; Nestadt et al., 2001; Bartz and Hollander, 2006; Murphy et al., 2013). Taken as a whole, these findings support the presence of convergent neuropathologies in anxiety and OCD (Nestadt et al., 2003), and suggest that it is critical to model both components of the disorder for a more accurate understanding of OCD pathophysiology. There is also substantial comorbidity between OCD and major depressive disorder (Nestadt et al., 2001, 2003; Denys et al., 2004c; Stein et al., 2010), further suggesting that the pathologic processes underlying OCD impact limbic circuitry. While obsessions and compulsions are not purely a product of emotional dysregulation, these observations support the notion that affective dysregulation–in particular pathologic anxiety–is of central importance to OCD.

### Corticolimbic-Ventral Striatal Models may Capture the Affective Components of OCD

In this review we focus on the role that converging inputs from a corticolimbic network to the VS may play in producing compulsive behaviors in OCD, with several important caveats in mind: (1) No model is a complete account of a neuropsychiatric disorder, and a corticolimbic-VS explanation of OCD does not capture all features of the disorder; (2) OCD is a heterogeneous disorder. Different symptom clusters may be produced by different neuronal or circuit abnormalities, and future work should address how the data and ideas covered here can be applied to different subtypes of OCD; (3) We focus on VS circuitry, with the understanding that other brain regions, including dorsal striatum, are clearly important to the pathophysiology of OCD, and that circuits including dorsal and VS are likely to be cooperative, and not antagonistic (Insel and Winslow, 1992; Graybiel and Rauch, 2000; Milad and Rauch, 2012; Burguière et al., 2013, 2015; Gremel and Costa, 2013; Gillan and Robbins, 2014; Pauls et al., 2014). Despite these caveats, investigating corticolimbic-VS circuitry and developing more expansive OCD models that take limbic information processing into account (Milad and Rauch, 2012; de Haan et al., 2013; Ahmari and Dougherty, 2015) are likely to help elucidate the pathophysiologic processes leading to OCD.

## Ventral Striatum as a Corticolimbic Integrator

### Ventral Striatal Cell Types and Connectivity

Over 90% of the neurons in the VS are medium spiny, γ-aminobutyric acid (GABA) projection neurons, and several types of interneurons comprise the remaining population (Kawaguchi et al., 1995; Kawaguchi, 1997; Meredith, 1999; Nicola et al., 2000; Kreitzer, 2009; Gerfen and Surmeier, 2011; Gittis and Kreitzer, 2012). Medium spiny neurons (also known as spiny projection neurons) are the main output neurons of the striatum, and reside in an intermingled pair of compartments, known as patch and matrix. These compartments are identified by distinct immunoreactivity and gene expression patterns, and medium spiny neurons tend to have dendritic arbors that are confined to their respective patch or matrix compartments (Graybiel and Ragsdale, 1978; Crittenden and Graybiel, 2011). These data suggest striatum follows complex organizational principles that are critical to understanding the function of this system. Striatal interneurons (fast-spiking parvalbumin positive, low-threshold spiking, calretinin positive, and cholinergic tonically active neurons) can powerfully influence the function of spiny projection neurons (Graybiel and Ragsdale, 1978; Kawaguchi et al., 1995; Kawaguchi, 1997; Tepper and Bolam, 2004; Humphries and Prescott, 2010; Crittenden and Graybiel, 2011). Some of these interneuron subtypes have been linked to OCD (Burguière et al., 2013) and Tourette’s syndrome (Xu et al., 2015) pathophysiology.

### Critical Features of VS Circuitry

The VS contains, and is often synonymous in the literature with, the NAc core and the NAc shell. Subsets of NAc core projection neurons may form direct and indirect pathways homologous to those seen in dorsal striatum, which have classically been thought to promote or suppress movements, respectively (Albin et al., 1989). However, it remains unclear how well this model applies to VS projections (Sesack and Grace, 2010; Kupchik et al., 2015). The NAc core direct pathway is formed by medium spiny neurons that predominantly express D1 dopamine receptors, and projects to the substantia nigra pars reticulata and VTA; the indirect pathway contains medium spiny neurons expressing D2 receptors, and projects to the ventral pallidum and subthalamic nucleus (Albin et al., 1989; Zahm and Brog, 1992; Graybiel, 2000; Zhou et al., 2003; Humphries and Prescott, 2010; Kravitz et al., 2010; Sesack and Grace, 2010; Freeze et al., 2013). It is more difficult to extrapolate the direct and indirect pathway model to the NAc shell, though some authors have argued that connections with regions not traditionally included in the classic pathways form direct and indirect pathways (O′Donnell et al., 1997; Nicola et al., 2000; Sesack and Grace, 2010; Kravitz and Kreitzer, 2012). As an alternative to conceptualizing the shell within the direct/indirect pathway framework, it can also be thought of as an extension of the amygdala complex on the basis of intermingled and overlapping projection targets, as well as cytoarchitecture (Zahm and Brog, 1992; Sesack and Grace, 2010; Zorrilla and Koob, 2013). Therefore, the NAc shell region is also part of a larger limbic network. Taken together, the connectivity of the NAc core and shell place the VS in a key position to integrate behavioral selection and affective information processing (Kelley, 2004; Balleine and O′Doherty, 2010; Volman et al., 2013).

**Figure 2:**
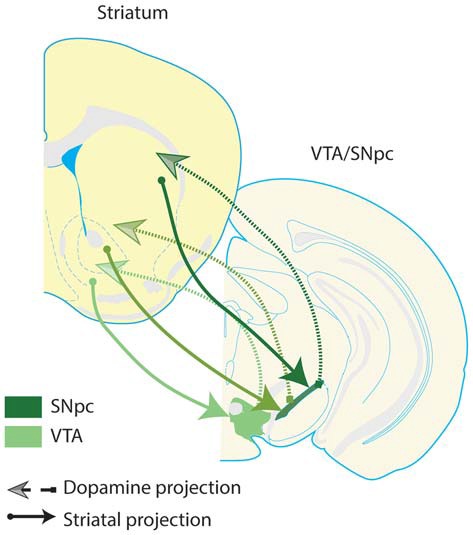
**Spiraling striatal-dopamine connections from ventral striatum to dorsal striatum.** Some striatal neurons project to dopaminergic regions of the midbrain, and dopamine neurons in turn innervate the striatum; there is a general ventral-to-dorsal topography of these connections. This figure presents a highly simplified schematic, depicting the ultimate flow of information from ventral to dorsal striatum, and from ventral to dorsal dopamine systems–VTA and substantia nigra pars compacta (SNpc). Arrows depicted in darker shades of green denote more dorsal pairs of striatal and dopamine projections. Note that dopamine regions receive input from a given striatal region and in turn, innervate a more dorsal region of the striatum. These connections suggest that there is a general ventral to dorsal flow of information through striatal-dopamine circuits, allowing information processed in VS to influence neuronal activity in the entire striatum, through a poly-synaptic pathway (Haber et al., 2000). This suggests that VS is able to integrate information transmitted by the corticolimbic inputs highlighted in this review into the dorsal striatum. Furthermore, these pathways provide one anatomical mechanism for a complementary relationship between the corticolimbic-ventral striatal model put forth here, and traditional cortico-striatal-thalamo-cortical circuit models of OCD. Dopamine neurons which innervate the same striatal region they receive input from are not depicted, but do exist.

Among the VS neurons that project to dopamine neurons, NAc shell neurons preferentially innervate dopamine neurons in the VTA, while NAc core neurons tend to innervate dopamine neurons in substantia nigra (Sesack and Grace, 2010). Subsets of substantia nigra and VTA dopamine neurons project to striatal subregions more dorsal to the striatal subregions they receive projections from Haber et al. (2000); Joel and Weiner (2000). This leads to a flow of information from ventral to dorsal striatal circuits via communication with dopamine neurons, and enables information processed within the VS to iteratively influence dorsal striatal computations (Figure 2).

### Corticolimbic Inputs Converge in Ventral Striatum

The VS inputs detailed in this review, transmit diverse information (Zorrilla and Koob, 2013), and the capacity of VS to integrate disparate information content is a defining feature of the region. Multiple cortical (Finch, 1996; Sesack and Grace, 2010) and amygdala (McGinty and Grace, 2009) inputs converge onto the same NAc neurons. In fact, up to 5-way convergence between afferent inputs to the VS can be demonstrated on single NAc neurons (Finch, 1996). Additional studies demonstrate basolateral amygdala and subiculum (French and Totterdell, 2003), and medial prefrontal cortex and subiculum (French and Totterdell, 2002), synaptic inputs on the same NAc neuron. Furthermore, NAc excitatory post-synaptic potentials evoked by basolateral amygdala stimulation are blocked by D1 dopamine receptor activation (Charara and Grace, 2003), suggesting that VS is an interface between dopamine and amygdala systems. Projections from the mOFC also terminate in the same VS regions as projections from VTA and the basolateral amygdala (Kelley et al., 1982; Ongur and Price, 2000; Voorn et al., 2004; Whiteside et al., 2004), though it is important to note that there is less direct evidence of convergence between amygdala and OFC inputs onto single NAc neurons. Because it is suggested that activation of NAc projection neurons is likely driven by nearly simultaneous co-activation of multiple inputs (Pennartz et al., 1994), converging inputs from these regions are likely to be essential for the VS to function as a limbic-behavioral interface (Mogenson et al., 1980).

### Converging Information in Ventral Striatum could Lead to OCD Symptomatology

By integrating several modalities of limbic information into striatal circuitry, the VS is a strong candidate for selection and promotion of compulsive behaviors in OCD patients. In support of this notion, the rodent VS has long been implicated in aspects of behavior that may play a role in, or reflect, compulsivity. These include reward, effort, cue-driven behavior, conditioning, and drug seeking (Parkinson et al., 1999; Robbins and Everitt, 2002; Salamone and Correa, 2002; Wise, 2004; Kalivas et al., 2005; Ikemoto, 2007; Yin et al., 2008; Carlezon and Thomas, 2009; Russo et al., 2010; Volman et al., 2013). Thus, a strong line of prior research suggests corticolimbic-VS networks may contribute to OCD symptomatology.

## Amygdala Signaling in the Ventral Striatum

### Amygdala-Ventral Striatum Circuit Anatomy

**Figure 3:**
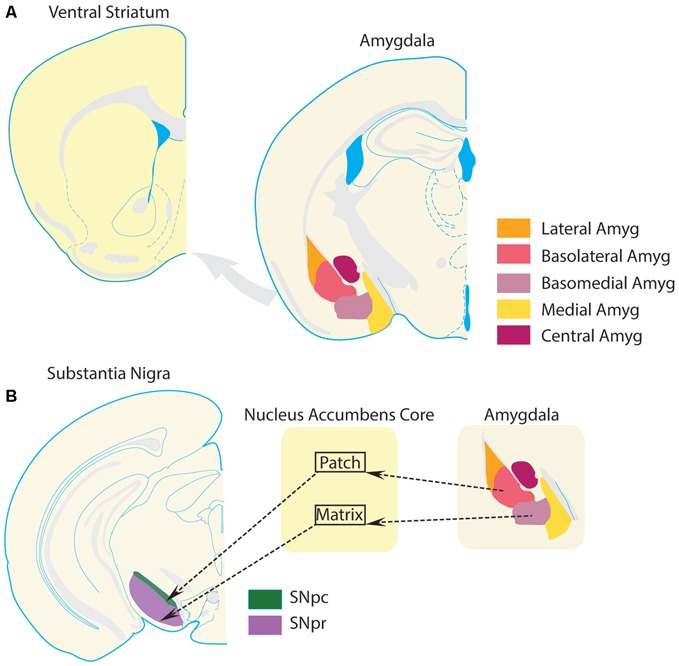
**Amygdala projections to ventral striatum.** The VS receives a glutamatergic projection from the basolateral amygdala complex. **(A)** Schematics of VS and amygdala following the same conventions as Figure 1 are presented for clarity. **(B)** Substantia nigra par compacta (SNpc), which receives input from projection neurons in the patch compartments of the NAc core, sends a large number of dopamine projections to the dorsal striatum. The substantia nigra pars reticulata (SNpr), which is part of the traditional striatal direct pathway and promotes selection of behaviors, is preferentially innervated by projection neurons in the matrix compartments of the NAc core. This compartmental segregation of NAc-SN projections is followed by the basolateral amygdala complex as well. Projections from the basolateral nucleus terminate preferentially in the patch compartments of NAc, while projections from the basomedial nucleus terminate preferentially in the matrix compartments of the NAc. Thus, a basolateral nucleus-NAc core patch- SNpc circuit could regulate dopamine transmission in the striatum. On the other hand, a basomedial-NAc core matrix- SNpr circuit could modulate information transmission in the direct pathway. Thus, these circuits provide a theoretical foundation for amygdala-VS circuits to modulate behavioral selection and striatal processing. Dysregulation of this circuit in OCD could potentially contribute to compulsive behavior.

The amygdala complex consists of several subregions, including the cortico-medial region (cortical, medial, and central nuclei), and basolateral region (Pitkänen et al., 1997; LeDoux, 2000). The basolateral region is often further subdivided according to several schemes, into lateral, basal, and accessory basal nuclei, or into lateral, basolateral, and basomedial nuclei. The basolateral subregion of the amygdala issues a dense glutamatergic projection to the VS. This projection is roughly topographical, such that rostral regions project to lateral VS (preferentially to NAc core), and caudal regions project more medially (preferentially to NAc shell; Kelley et al., 1982; Wright et al., 1996; Pitkänen et al., 1997; LeDoux, 2000; Voorn et al., 2004; Sesack and Grace, 2010; Janak and Tye, 2015). Projections from the basolateral amygdala to the NAc core also tend to follow compartmental boundaries (Figure 3). Specifically, basal nucleus projections largely terminate in NAc core patches (Wright et al., 1996). Since dopaminergic neurons preferentially receive input from medium spiny neurons that are located in the patch compartments, including those dopamine neurons projecting to more dorsal segments of the striatum (Berendse et al., 1992; Wright et al., 1996; Crittenden and Graybiel, 2011), basal amygdala inputs to the NAc core could influence information processing throughout the entire striatum and dopamine system. In contrast, amygdala accessory basal nucleus projections preferentially terminate in the matrix compartments of the core (Wright et al., 1996). Many NAc core matrix neurons project to the substantia nigra pars reticulata, an output nucleus of the basal ganglia, and therefore could contribute directly to behavioral selection (Berendse et al., 1992; Wright et al., 1996). Thus, the VS serves as a region in which limbic information from the amygdala can directly impact behavior and modulate the flow of information throughout the entire striatum.

### Amygdala Processes Multiple Forms of Affective Information

The amygdala facilitates appropriate behavioral responses to aversive or harmful stimuli (Davis, 1992; LeDoux, 2000), and has classically been associated with fear (also conceptualized as threat) conditioning and extinction (LeDoux, 2000, 2007, 2014). According to simplified models of fear/threat conditioning in rodents, multimodal, threat-related, sensory information from cortical and thalamic projections is transmitted directly to the lateral nucleus of the amygdala (LeDoux et al., 1990), and is subsequently transferred to other basolateral nuclei. Information then flows from the basolateral nuclei to the central nucleus of the amygdala, which functions as an output to numerous brain regions (LeDoux, 2007; Janak and Tye, 2015). Lesions of the basolateral amygdala disrupt acquisition of conditioned fear (Nader et al., 2001), and central amygdala is commonly associated with expression of conditioned fear (Hitchcock and Davis, 1986). Therefore, the amygdala is critical for threat learning and promoting behaviors driven by fearful situations (LeDoux, 2014).

In addition to a role in fear and threat conditioning, the amygdala also regulates anxiety. The activity of basolateral amygdala networks reflects the anxiogenic potential of the environment, and points to a role for this subregion in processing anxiety-related information (Likhtik et al., 2014). Recent reports also suggest that subsets of intra-amygdala projections bidirectionally modulate anxiety-like behavior (Tye et al., 2011; Janak and Tye, 2015). Finally, the basolateral amygdala also contributes to anxiogenic effects through connections with the ventral hippocampus (Felix-Ortiz et al., 2013). These findings detail some of the circuits of the basolateral amygdala that contribute to anxiety-related behaviors. Though these projections have not been fully mapped, and their effects on information processing in VS are not completely understood, it is clear that interconnected basolateral amygdala networks modulate anxiogenic and anxiolytic states. These same networks could convey anxiety-related information to VS, which could directly impact behavioral selection and reinforcement processes important to OCD.

Early amygdala lesion studies suggested that amygdala is involved in numerous aspects of cognition, and is not limited to processing aversive information (Zorrilla and Koob, 2013; Janak and Tye, 2015). Specifically, basolateral amygdala, and its projections to the VS, are necessary for some forms of reward-related Pavlovian and instrumental behavior (Cador et al., 1989; Everitt et al., 1989; Hiroi and White, 1991; Hatfield et al., 1996; Setlow et al., 2002; Stuber et al., 2011), and neurons in the basolateral amygdala also respond to rewarding stimuli (Schoenbaum et al., 1999; Paton et al., 2006; Morrison and Salzman, 2010), indicating that the same regions that drive fear learning also contribute to appetitive learning. A growing appreciation for the complexity of the amygdala complex has resulted in a more nuanced understanding of this region’s role in fear learning, appetitive learning, and value encoding (Janak and Tye, 2015), and it is clear that the amygdala promotes adaptive behavioral responses to both positive and negative environmental stimuli.

### Dysregulation of Emotional Information Processing in Amygdala could Contribute to Compulsivity

By transmitting information related to anxiety, fear, and reward to the VS, the amygdala regulates how affect and motivation influence adaptive behavior. As mentioned above, compulsivity is often driven by anxiety, threat avoidance, or aberrant motivation in OCD patients (Milad and Rauch, 2012; Pauls et al., 2014; Ahmari and Dougherty, 2015). Since the amygdala is central to processing these forms of information (Janak and Tye, 2015), aberrant communication of anxiety-related information from amygdala to VS could mediate anxiety-driven compulsive behavior. Likewise, active threat avoidance behavior requires intact basolateral amygdala and VS (Bravo-Rivera et al., 2014), suggesting that activity in this circuit could lead to excessive avoidance behavior in OCD. Collectively, this suggests that amygdala is central to multiple aspects of cognition that are impaired in OCD, and that amygdala dysregulation may contribute to compulsivity by imparting excessive affective influence on behavioral selection.

In support of this theory (though less commonly highlighted in the literature), structural and functional amygdala abnormalities are associated with OCD. For instance, decreased amygdala volume has been shown in OCD patients (Szeszko et al., 1999; Pujol et al., 2004). Functional imaging studies have also suggested that amygdala representations of neutral or positive valences may be blunted in OCD. Specifically, when OCD patients view neutral faces (Cannistraro et al., 2004; Britton et al., 2010) or are anticipating rewards (Marsh et al., 2015), amygdala is hypoactive compared to controls (but see Simon et al., 2014). In contrast, amygdala is hyperactive in patients viewing images of fearful expressions; the degree of activation is correlated with symptom severity (Via et al., 2014). Likewise, threatening stimuli (Admon et al., 2012), and symptom provoking stimuli (Breiter et al., 1996; Mataix-Cols et al., 2004; van den Heuvel et al., 2004; Simon et al., 2010, 2014; Milad and Rauch, 2012) overactivate the amygdala of OCD patients. It should be noted, however, that these findings have not been uniformly replicated (Cannistraro et al., 2004). While it is unclear what underlies these discrepancies in the literature, a substantial amount of data suggest that threatening, fearful, and symptom provoking stimuli are over-represented in amygdala in OCD.

All told, these data suggest that the amygdala can be either hyperactive or hypoactive in patients with OCD. This could lead to low signal-to-noise representations of neutral or positive affective information. In other settings, such as during symptom provocation or when dealing with threatening stimuli, amygdala could be overactive and produce excessive emotional processing in VS. These disruptions could lead to weakened representations of neutral stimuli (for example, items in a room), but excessively strong representations of symptom provoking stimuli (for example, a trashcan or a messy bathroom). The effect of this imbalance in representational strength could ultimately lead to selection of behaviors in striatal circuits on the basis of disproportionately strong emotional representations. In this example, the sight of a trashcan or bathroom mess could provoke cleaning and sanitation compulsions. Thus, through connections with the VS, the amygdala may play a critical role in OCD symptoms.

## Ventral Tegmental Area Signaling in the Ventral Striatum

### Ventral Tegmental Area Anatomy

Preclinical anatomical studies indicate that the VTA is a functionally and anatomically heterogeneous structure which contains a pair of major nuclei, the parabrachial and paranigral, and is often described as containing several smaller nuclei, including the central linear, rostral linear, and interfasicular nuclei (Oades and Halliday, 1987; Ikemoto, 2007; Sanchez-Catalan et al., 2014). Approximately two thirds of the neurons in the VTA are dopaminergic, and the majority of the remaining neurons are GABAergic (Swanson, 1982; Nair-Roberts et al., 2008; Sesack and Grace, 2010). While there are local GABAergic connections in the VTA, many VTA GABA neurons are projection neurons (Van Bockstaele and Pickel, 1995; Carr and Sesack, 2000; Omelchenko and Sesack, 2009). In recent years, it has come to light that VTA also contains a sizeable population of neurons that release glutamate, and that dopamine neurons can co-release glutamate and GABA (Yamaguchi et al., 2007, 2011; Hnasko et al., 2010; Stuber et al., 2010; Tritsch et al., 2012). Thus, the VTA projects a chemically diverse signal to regions such as the prefrontal cortex, amygdala, and VS (Swanson, 1982; Van Bockstaele and Pickel, 1995; Carr and Sesack, 2000). The various VTA projections are intermingled and have differing electrophysiological properties and protein expression patterns, largely along a mediolateral gradient (Lammel et al., 2008, 2014; Volman et al., 2013). These anatomical characteristics enable the VTA to subserve a diverse assortment of cognitive functions in downstream brain regions.

### Dopaminergic Signaling in Ventral Striatum Directly Contributes to Behavioral Selection

The VS receives a dense dopaminergic projection from the VTA, and a much less studied GABAergic projection (Swanson, 1982). The firing patterns of dopamine neurons, including those projecting to the VS, encode errors in the predicted value of rewards (Ljungberg et al., 1992; Schultz et al., 1993, 1997; Montague et al., 1996; Schultz, 1998). This signal establishes learned associations and guides behavioral selection (Houk et al., 1995; Montague et al., 1996; Schultz et al., 1997; Schultz, 1998; Waelti et al., 2001; Joel et al., 2002; Morris et al., 2006; Roesch et al., 2007; Glimcher, 2011). Moreover, this projection subserves various aspects of reinforcement and motivation (Schultz, 1998; Wise, 2004; Salamone et al., 2007). Thus, VTA innervation of VS strongly modulates and sustains behavioral output.

Dopamine is released in the VS in response to rewarding or reward-predictive stimuli, including food and drugs of abuse (Phillips et al., 2003; Roitman et al., 2004, 2008; Stuber et al., 2005; Brown et al., 2011; McCutcheon et al., 2012), and often decreases the activity of VS medium spiny neurons (Peoples and West, 1996; Cheer et al., 2005; Roitman et al., 2005; Wheeler and Carelli, 2009). Some studies, however, have reported that subsets of VS projection neurons are also activated by aversive stimuli (Setlow et al., 2003; Roitman et al., 2005; Kravitz and Kreitzer, 2012), which highlights the heterogeneity of striatal representations. Taken together, these data suggest that the activity of VS medium spiny neurons is strongly modulated by dopaminergic input, which contributes to behavioral selection and value representation.

### Multiple Lines of Clinical Evidence Suggest that OCD is Characterized by a Hyperdopaminergic State

There are several lines of evidence for dopamine system pathology in OCD patients. Several studies have reported reduced D1 and D2 dopamine receptor binding in striatum (Denys et al., 2004b; Hesse et al., 2005; Nikolaus et al., 2010; Klanker et al., 2013), including in the VS (Figee et al., 2014), which is hypothesized to be a compensatory response to increased dopaminergic tone (Klanker et al., 2013). These findings go hand-in-hand with several pharmacological findings. For instance, cocaine, amphetamine, and methylphenidate, which are indirect dopamine agonists, can induce or exacerbate OCD symptoms (Denys et al., 2004a). When dopamine antagonists are used to augment selective serotonin reuptake inhibitors, symptom improvement is sometimes observed; this is especially the case in patients with comorbid tic disorder (McDougle et al., 2000; Denys et al., 2004a; Klanker et al., 2013). Thus, abnormalities in the dopamine system could account for some of the dysfunction observed in OCD.

Abnormalities in the gene encoding catechol-o-methyl-transferase (COMT), which metabolically terminates dopamine signaling following neurotransmitter release, also suggest that dopamine signaling may be disrupted in patients with OCD. A functional allele of COMT that is linked to decreased metabolism of dopamine is associated with susceptibility to OCD (Karayiorgou et al., 1997, 1999; Alsobrook et al., 2002). Additionally, there are significant differences between distributions of COMT high activity homozygotes, low activity homozygotes, and heterozygotes in OCD patient and control populations (Schindler et al., 2000; Niehaus et al., 2001). It should also be noted that several studies have found no association between this COMT allele and OCD patients (Denys et al., 2004a); however, the limited evidence available suggests that COMT could be hypofunctional in OCD, and may ultimately lead to hyperdopaminergic signaling (Denys et al., 2004a; Klanker et al., 2013).

While a substantial proportion of the available evidence suggests that OCD is associated with a hyperdopaminergic state, there are also findings that are generally suggestive of a more complex relationship between OCD symptomatology and dopamine. For instance, in spite of the above findings suggesting that excessive dopamine signaling may contribute to pathology in OCD, there is: (1) evidence that dopamine antagonism can also exacerbate symptoms or fail to produce a clinical benefit (Denys et al., 2004a; Klanker et al., 2013; Simpson et al., 2013); and (2) imaging data suggesting both increased and decreased dopamine transporter binding (Denys et al., 2004a; van der Wee et al., 2004; Hesse et al., 2005; Klanker et al., 2013). These inconsistencies suggest that unidirectional disruptions of dopaminergic signaling may not provide a straightforward explanatory model of OCD. We therefore theorize that OCD may be associated with a trend towards excessive dopaminergic signaling, with the understanding that this literature is evolving, and must account for some inconsistencies moving forward.

### Relationship between Dopamine Signaling and Repetitive Behaviors in Animal Models

One approach to clarifying the role of dopamine signaling in OCD is to employ animal models and systematically probe the function of the dopamine system. There is clear evidence that modulating dopamine signaling in the striatum leads to abnormal repetitive behaviors in animals. Knockdown of the dopamine transporter leads to excessive grooming in rodents (Berridge et al., 2005), and D1 agonists and partial agonists also produce a similar effect (Molloy and Waddington, 1987; White et al., 1988). Likewise, when amphetamine is injected directly into the VS, it produces stereotyped and repetitive behaviors (Colle and Wise, 1991). Quinpirole, a D2/D3 dopamine receptor agonist, has also been used to model compulsive behaviors associated with OCD, and electrical stimulation of the VS alleviates the increased frequency of these abnormal behaviors (Einat and Szechtman, 1995; Joel, 2006; Mundt et al., 2009). Thus, dopaminergic manipulations produce repetitive behaviors that may be used to model some aspects of compulsivity in OCD patients. While these manipulations do not reproduce all aspects of the disorder, they are important tools for understanding the neuronal underpinnings of some of the behavioral symptoms seen in OCD.

All told, dopaminergic inputs to the VS likely contribute to selecting and sustaining appropriate behavioral sequences, in part through representations of action value and through reinforcement processes. A hyperdopaminergic state, however, could lead to excessive valuation of a behavior, or repetitive selection of a behavior. With this in mind, dopamine signaling in the VS is well positioned to directly contribute to OCD-related compulsivity. VTA projections to the VS may be particularly relevant to OCD because this pathway is classically associated with producing and sustaining effortful behaviors, mediating locomotor arousal, and energizing behavioral responses (Salamone and Correa, 2002; Wise, 2004; Salamone et al., 2007). Thus, through several mechanisms, dysregulation of this circuitry could contribute to compulsive behavior.

## Orbitofrontal Cortex Signaling in the Ventral Striatum

### Orbitofrontal Cortex Projections

The OFC receives a wide variety of inputs from sensory structures, the subiculum, entorhinal cortex, and amygdala, so that the external world is richly represented and integrated with contextual, limbic, and sensory information (Krettek and Price, 1977; Groenewegen et al., 1997; Ongur and Price, 2000). Rodent prefrontal regions are identified on the basis of thalamic projections (Rose and Woolsey, 1948; Leonard, 1969; Uylings et al., 2003). Thus, the lateral orbitofrontal, ventrolateral orbitofrontal, and agranular insular cortices are all considered lateral OFC (Ongur and Price, 2000; Zald and Rauch, 2006; Price, 2007), while the medial OFC and ventromedial OFC comprise the mOFC. It is important to clarify that the term “medial OFC” can be used to refer to a specific subregion in the prefrontal cortex, or can refer to the pair of aforementioned regions. Throughout this review the term is used in the latter sense. Based on connectivity, the mOFC regions bear a stronger resemblance to other medial prefrontal cortex regions, such as infralimbic and prelimbic cortex, than to the lateral OFC (Zald and Rauch, 2006; Price, 2007). Specifically, the mOFC preferentially innervates the VS, while lateral OFC innervates more dorsal components, particularly the centromedial striatum (Ongur and Price, 2000; Price, 2007; Schilman et al., 2008; Hoover and Vertes, 2011; Rodriguez-Romaguera et al., 2015). The striatal projection patterns of lateral OFC and mOFC thus implicate these structures in distinct cognitive operations.

### Medial Orbitofrontal Cortex Regulates Behavioral Selection and Value-Based Information Processing

The mOFC is closely linked to outcome-based behavioral selection. A thorough meta-analysis of 87 functional imaging studies in humans revealed an association between mOFC activation and positive valence stimuli, such as reward (Kringelbach and Rolls, 2004). This region is also implicated in evaluating and choosing between actions, as demonstrated by the fact that mOFC lesions impair the ability of non-human primates to select the most advantageous option when comparing differently-valued outcomes (Rudebeck and Murray, 2011; Milad and Rauch, 2012; Noonan et al., 2012). Further implicating mOFC in choosing between behavioral options are lesion studies in rodents which demonstrate excessive risky behavioral choices (Stopper et al., 2014). Finally, in support of the idea that mOFC encodes information governing behavioral selection, a meta-analysis of human imaging studies suggests that mOFC represents a common neuronal currency for rewards and punishments, so that different modalities of value are encoded in the same networks (Levy and Glimcher, 2012). These observations suggest that mOFC could subserve decision making by integrating information about the outcomes that could result from an animal’s competing behavioral options (Rudebeck and Murray, 2014). Congruent with this view, mOFC has been implicated in selecting and adapting goal-directed behaviors and transforming value representations into behavior (Hollerman et al., 2000; Balleine and O′Doherty, 2010; Gourley et al., 2010). Thus, mOFC dysregulation could contribute to OCD symptoms by encoding aberrant representations of value comparisons between behaviors, leading patients with OCD to engage in compulsive behaviors despite negative consequences.

### Medial OFC is Structurally and Functionally Altered in OCD

Before discussing OFC abnormalities in OCD patients, it is important to note that some studies do not clearly differentiate between medial and lateral OFC, and thus focusing exclusively on mOFC in the human imaging literature can be challenging. OCD is generally associated with hyperactivity in OFC (Saxena et al., 2001; Whiteside et al., 2004), and a meta-analysis of imaging studies confirmed that abnormalities in OFC activity are consistently observed in OCD patients (Menzies et al., 2008). Early functional imaging studies reported increased activity in bilateral OFC (Alptekin et al., 2001), left OFC (Swedo et al., 1989), and orbital gyrus (Baxter et al., 1988) of OCD patients at rest; the magnitude of OFC metabolic activation was correlated with clinical ratings of symptom severity (Swedo et al., 1989). Supporting suggestions that OFC hyperactivity may lead to obsessions and compulsions, symptom provocation in OCD patients increases bilateral OFC metabolic activity (Rauch et al., 1994). Furthermore, left OFC activity is increased relative to resting state when OCD patients, but not healthy controls, are exposed to stimuli designed to evoke feelings of disgust (Stein et al., 2006). Thus, most evidence suggests that increased OFC activity is a component of OCD pathophysiology (Saxena et al., 2001). In support of this idea, selective serotonin reuptake inhibitors, which are first-line treatments for OCD, decrease OFC metabolic activity; activity in both lateral and mOFC is more strongly reduced in treatment responsive patients than non-responsive patients (Swedo et al., 1992; Saxena et al., 1999, 2002).

However, hyperactivity in the OFC of OCD patients has not been uniformly reported. Functional imaging studies have reported decreased OFC metabolic activity during several cognitive tasks. For example, during a stop signal task, which measures inhibitory control, adolescents with OCD show decreased metabolic activity in right OFC vs. healthy controls (Woolley et al., 2008). During reversal learning tasks, metabolic activity in lateral OFC and mOFC is also reduced in patients with OCD and their family members, but not healthy controls (Remijnse et al., 2006; Chamberlain et al., 2008). Further mechanistic studies in both humans and animals are needed to relate hyperactivity at rest and during symptom provocation (Insel and Winslow, 1992; Saxena et al., 2001; Nakao et al., 2005; Milad and Rauch, 2012; Ahmari and Dougherty, 2015) with these reports of hypoactivation during cognitive tasks. One potential explanation is that OFC representations may have decreased signal-to-noise ratios in OCD patients (greater activity levels at baseline and blunted activation when the region is engaged). This could ultimately lead to improper value-based behavioral selection, and potentially contribute to the symptoms manifested in OCD.

OCD is also generally associated with structural OFC abnormalities–specifically decreased OFC volume, including in mOFC, compared to healthy controls (Szeszko et al., 1999; Pujol et al., 2004; Rotge et al., 2009). Furthermore, treatment-refractory patients have decreased OFC volume compared to treatment-responsive cases (Atmaca et al., 2006). These findings, however, have not been uniformly replicated (Kim et al., 2001); several factors may underlie this contradiction (for further details, see Ahmari, 2015). Because this review focuses on interregional communication with the VS, reports of increased structural and functional connectivity between mOFC and VS are of particular interest (Sakai et al., 2011; Nakamae et al., 2014). This increased connectivity significantly predicts YBOCS score (Harrison et al., 2009), strongly suggesting a relationship between signaling in these regions and OCD symptoms. However, care must be taken interpreting these results, as a recent study found a significant negative correlation between VS and mOFC connectivity and YBOCS score (Posner et al., 2014). In total, these findings suggest that patients with OCD have both aberrant OFC activity and altered anatomy and connectivity with other brain regions.

These data support the idea that OFC dysregulation may produce abnormal repetitive behaviors, in part through mOFC signaling in the VS. Potential mechanisms include aberrant processing within mOFC leading to corrupted representations of value related information, which the VS utilizes for action selection. A second complementary idea is that mOFC activity in OCD patients could preferentially drive direct pathway VS neurons, ultimately leading to aberrant and repetitive behavioral output (Pauls et al., 2014). Although OFC control over striatal production of compulsive behaviors is classically associated with dorsal regions of the striatum (Fineberg et al., 2010; Albelda and Joel, 2012), the data summarized above suggest a more complex view, by strongly implicating the mOFC and its VS connections in the production of OCD symptoms.

## Corticolimbic-Ventral Striatum Networks Involved in the Production of Compulsive Behaviors

In the final section of this review, we will discuss the role of corticolimbic-VS networks in instrumental and Pavlovian behaviors, and explore the relationship of these behaviors to OCD. We then theorize how dysfunction in this network could contribute to compulsive behaviors in OCD. Last, we discuss how the VS may be both a locus of pathology and site of effective intervention in OCD.

### Goal-Directed, Habitual, and Pavlovian Behaviors

Behaviors can be classified according to the relationship between an action and the resulting outcome of that action. Instrumental behaviors, which require execution of an action in order to earn an outcome, can be either goal-directed or habitual. Goal-directed behaviors are those that are selected according to a representation of the contingency (the causal relationship) between the action and the resulting outcome, and the value of the outcome. An animal will stop selecting a goal-directed behavior if the outcome is devalued, or if the contingency between the action and outcome is degraded. In contrast, habitual behaviors are highly automatized behaviors that are triggered by stimuli in the environment, despite devaluation of the outcome, or degradation of the contingency between actions and outcomes (Balleine and Dickinson, 1998; Daw et al., 2005; Yin et al., 2008; de Wit and Dickinson, 2009). Pavlovian behaviors are elicited by a stimulus predicting an outcome; the animal’s performance of the behavior does not cause outcome delivery (Balleine and Dickinson, 1998; Yin et al., 2008; de Wit and Dickinson, 2009). Distinct neural mechanisms may contribute to these three forms of behavioral selection.

### Compulsive Behaviors in OCD may be Mediated by Several Selection Mechanisms

Compulsive behaviors in OCD are an example of how neuropathological processes can subvert behavioral selection, and how dysregulation of behavioral selection may contribute to debilitating symptomatology. The specific mechanisms involved are an open question, as it is unknown to what extent compulsive behaviors in OCD are selected by habit-like mechanisms (Graybiel and Rauch, 2000; Gillan and Robbins, 2014; Burguière et al., 2015), goal-directed mechanisms (McFall and Wollersheim, 1979; Rachman, 1997), or both (Piantadosi and Ahmari, 2015). Several lines of evidence also suggest that Pavlovian processes may contribute to OCD symptomatology (Milad et al., 2013; McLaughlin et al., 2015). Thus, multiple behavioral selection processes could contribute to compulsive symptoms (Sjoerds et al., 2014; Piantadosi and Ahmari, 2015). The corticolimbic-VS network is well positioned to modulate aspects of habitual, goal-directed, and Pavlovian behavior in OCD, as discussed further below.

### The Corticolimbic-Ventral Striatal Network Subserves Performance of Goal-Directed Behavior

The VS guides performance of goal-directed behaviors (Cardinal et al., 2002a; Yin et al., 2008; Hart et al., 2014), and the importance of this region is most apparent when greater levels of motivation or behavioral organization are required. For instance, while NAc core lesions do not disrupt performance of actions resulting in immediate delivery of outcomes, acquisition is impaired when reinforcement is delayed (Cardinal et al., 2002b; Cardinal and Cheung, 2005). Additionally, depleting dopamine levels in the NAc selectively impairs behavioral performance when large numbers of actions are required (Aberman and Salamone, 1999). These findings suggest that VS, in conjunction with VTA dopamine neurons, encodes information critical for performing behavior across delays in time and until behavioral requirements are fulfilled. Thus, the VS facilitates and sustains performance of complex goal-directed behaviors.

**Figure 4:**
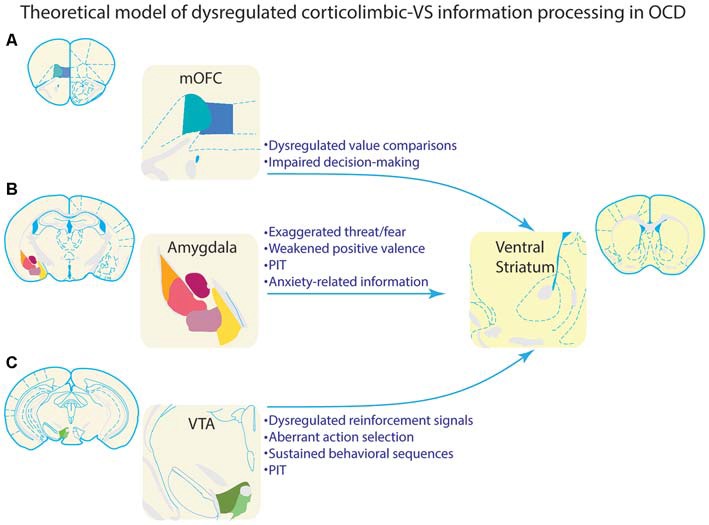
**Theoretical model of how dysregulated information processing in corticolimbic inputs to ventral striatum could underlie compulsive behavior in OCD.** Each of the corticolimbic inputs highlighted in this review are depicted in the left column. VS is depicted in the right column for illustrative purposes. Bulleted text highlights information that is processed by each input region, and could theoretically be a source of dysregulated information processing in VS of OCD patients. **(A)** Value comparisons and decision-making are important functions of mOFC. Aberrant representations of this information could lead individuals with OCD to improperly evaluate behavioral choices. This could, in turn, lead to inflated valuation, and subsequent selection of compulsive behaviors. Alternatively, these aberrant representations could render goal-directed decision making ineffective, and bias behavioral control toward habit-like selection of compulsive behaviors. **(B)** The amygdala processes threatening/fearful stimuli, rewarding stimuli, and aversive stimuli. Aberrant representations of this information in OCD could lead to improper behavioral selection and compulsive behavior. As summarized in the text, amygdala is often hypoactive in response to rewarding or neutral stimuli, and hyperactive in response to threatening or symptom-provoking stimuli, which could lead to excessive influence of these stimuli over behavior. This is consistent with clinical observations of provocation of compulsive behaviors by environmental stimuli that are perceived as threatening. Amygdala projections to VS are particularly important to Pavlovian instrumental transfer (PIT). Symptom provoking stimuli could theoretically acquire an influence over behavior through PIT, and thus, dysregulation of PIT could also contribute to OCD. Amygdala is also important for processing anxiety, which is associated with the generation and/or maintenance of compulsive behaviors in OCD; this link places special emphasis on amygdala-based information processing in OCD. **(C)** The VTA is a major source of dopamine input to VS, and processes reinforcement signals which inform behavioral selection. This input is also necessary for sustaining behavioral sequences and effortful behavior. Considerable evidence suggests that OCD is associated with dysregulation of the dopamine system, potentially leading to a hyperdopaminergic state. These disruptions would theoretically impact information processing necessary for selecting and sustaining behavioral sequences, and could promote compulsive behavioral patterns. VTA input to VS is also necessary for PIT, and a hyperdopaminergic state could also contribute to the ability of environmental stimuli to impact behavioral selection in OCD.

While some earlier work suggested that NAc does not support sensitivity to outcome value (Balleine and Killcross, 1994), lesions of the NAc core cause animals to respond equivalently for devalued and non-devalued outcomes (Corbit et al., 2001), suggesting that NAc core facilitates selective outcome value-based performance. Disconnection of basolateral amygdala and NAc core also produces deficits in selective outcome value sensitivity (Shiflett and Balleine, 2010). As noted above, medial orbitofrontal lesions impair value comparisons between different outcomes (Noonan et al., 2010). These findings suggest that the VS, in conjunction with the mOFC, dopamine system, and basolateral amygdala, could underlie outcome-value comparisons, behavioral selection, and performance of goal-directed behavioral choice (Figure 4).

### Theoretical Contributions of Corticolimbic-Ventral Striatal Dysregulation to Habitual and Goal-Directed Behaviors

Habitual behaviors often emerge when regions supporting goal-directed behaviors are compromised (Balleine and Dickinson, 1998; Killcross and Coutureau, 2003; Daw et al., 2005; Yin et al., 2005, 2006; Yin and Knowlton, 2006; Gruner et al., 2015). As discussed in the preceding sections, OCD is associated with many examples of disrupted information processing (Figure 4) in networks supporting goal-directed behavior, which could potentially bias behavior toward habit-like selection via improper or ineffective evaluation and representations of actions and outcomes (Gruner et al., 2015). Consistent with this idea, OCD patients form habits more readily than healthy controls (Gillan and Robbins, 2014; Gillan et al., 2014, 2015). VS connections with dopamine neurons projecting to dorsal striatum could form a polysynaptic pathway to dorsal striatal regions commonly associated with habitual behavior. Through this pathway, dysregulation in VS could theoretically contribute to habit-like production of compulsive behaviors in OCD.

Damage to the VS also alters goal-directed decision-making (Floresco et al., 2008). For instance, NAc core lesions cause rats to make risk-averse choices (Cardinal and Howes, 2005) and prefer smaller rewards at shorter delays (Cardinal et al., 2001; Mar et al., 2011). Compromised VS information processing in patients with OCD could therefore impair or alter decision-making processes. In turn, this could lead to goal-directed selection of compulsive behaviors, if the patient believes their compulsions are either less risky (because of beliefs that performing the compulsion will prevent the outcome associated with the obsessive thought), or more likely to lead to immediate reward (because performing the compulsion leads to immediate anxiety relief). The basic premise of these notions is consistent with cognitive and goal-directed models of compulsive behavior in OCD (Piantadosi and Ahmari, 2015). Synthesizing these points with those mentioned in the preceding paragraph suggests that disrupted VS activity could theoretically lead to selection of compulsive behaviors via either habit-like or goal-directed mechanisms.

### The Corticolimbic-Ventral Striatal Network Underlies Pavlovian Influences on Behavior

Strong evidence shows that VS is essential for Pavlovian conditioning (Cardinal et al., 2002a; Yin et al., 2008), including studies demonstrating that lesions of the NAc core impair Pavlovian autoshaping (Parkinson et al., 2000), and produce deficits in reestablishing pre-lesion Pavlovian approach (Parkinson et al., 1999). As discussed below, Pavlovian learning requires converging limbic inputs from amygdala and VTA to the VS, which emphasizes the importance of interregional signaling for the role of VS in cognition (Kelley, 2004).

The VTA is particularly important in this regard, because it is the main source of dopamine for the VS. Several lines of evidence highlight the contributions of this projection system to Pavlovian behavior. First, dopamine receptor blockade disrupts acquisition and performance of conditioned approach to stimuli associated with reward (Di Ciano et al., 2001). Similarly, lesioning dopaminergic inputs to the NAc with 6-hydroxydopamine impairs acquisition of Pavlovian approach behavior (Parkinson et al., 2002), and post-training infusion of D1 dopamine receptor antagonists blocks consolidation of Pavlovian associations (Dalley et al., 2005). Taken together, this suggests that VS and the VTA dopaminergic neurons that project to the VS form part of a network mediating Pavlovian conditioning.

The amygdala also serves as an ideal candidate to support Pavlovian conditioning, because disconnecting basolateral amygdala and VS via contralateral lesions disrupts performance of conditioned place preference (Everitt et al., 1991), and impairs both autoshaping (Chang et al., 2012) and second order conditioning (Setlow et al., 2002). Thus, the basolateral amygdala’s connections to the VS also strongly contribute to Pavlovian conditioning.

Pavlovian associations can influence performance of instrumental behaviors via the process of Pavlovian to instrumental transfer (PIT), which occurs when presentation of a conditioned stimulus associated with reward increases instrumental responding (Estes, 1948). Lesion studies demonstrate that NAc core and shell support PIT (Corbit et al., 2001; Corbit and Balleine, 2011). Dopamine bidirectionally modulates PIT, as VTA inactivation attenuates PIT (Murschall and Hauber, 2006; Corbit et al., 2007), and intra-accumbens microinjections of amphetamine, an indirect dopamine agonist, enhance PIT (Wyvell and Berridge, 2000). Amygdala projections to the VTA also contribute to PIT (Corbit and Balleine, 2005; Shiflett and Balleine, 2010). Finally, OFC lesions decrease PIT, though it is unclear if this effect generalizes to both medial and lateral OFC (Ostlund and Balleine, 2007a,b; Balleine et al., 2011). Thus, the combined network of VS, amygdala, VTA, and OFC produce a motivational effect of Pavlovian associations on performance of instrumental behaviors.

### Dysregulation of Corticolimbic-Ventral Striatal Networks could Promote Pavlovian Influences on Compulsive OCD Symptomatology

It is critical to note the impact of Pavlovian motivational influences on behavioral selection, because abnormally high motivation to select a behavior may be a critical feature of compulsivity (Sjoerds et al., 2014; Piantadosi and Ahmari, 2015). For instance, symptom-provoking stimuli may have excessively strong conditioned associations with aversive outcomes (Simon et al., 2010; Ludvik et al., 2015). These stimuli could, in turn, bias behavioral selection and motivate compulsive behavior through PIT of aversive associations, or Pavlovian fear conditioning-like processes (Figure 4). The abnormally strong amygdala activation or hyperdopaminergia that may occur in OCD patients (Breiter et al., 1996; Denys et al., 2004a; Mataix-Cols et al., 2004; van den Heuvel et al., 2004; Simon et al., 2010, 2014; Milad and Rauch, 2012; Klanker et al., 2013) could underlie this motivational influence on behavioral selection. This notion is in line with previous suggestions that the corticolimbic regions addressed in this review process affect and value, and inform behavioral selection in VS (Mogenson et al., 1980; Cardinal et al., 2002a; Wise, 2004; Sesack and Grace, 2010; Zorrilla and Koob, 2013). Further support for aberrant Pavlovian information processing in OCD patients is provided by recent findings of abnormal Pavlovian fear extinction in patients with OCD (Milad et al., 2013; McLaughlin et al., 2015). Consistent with the idea that Pavlovian processes could underlie symptomology, extinguishing negative conditioned associations via exposure to symptom provoking stimuli has proven efficacious in OCD patients (Foa et al., 2005; Franklin and Foa, 2011; Simpson et al., 2013; Lewin et al., 2014; Ludvik et al., 2015).

## Predictions of Corticolimbic-Ventral Striatal Model

We have discussed the evidence suggesting that affective dysregulation is a key component of OCD, as well as evidence indicative of amygdala, OFC, and VTA pathologies in OCD. We also highlighted theoretical interregional circuit level mechanisms that could mediate or contribute to compulsive behavioral selection. These points lead to at least three additional predictions: (1) functional and structural abnormalities in VS should be present in patients with OCD; (2) a causal relationship should exist between corticolimbic-VS hyperactivity and compulsive-like behaviors in animal models; and (3) interventions which target the VS should ameliorate both affective and compulsive symptoms. The remainder of this review will explore these points below.

### Prediction 1: Structural and Functional Abnormalities in Ventral Striatum are Observed in Patients with OCD

In support of this prediction, non-medicated OCD patients have increased local connectivity in the VS compared to medicated patients and healthy controls (Beucke et al., 2013). As assessed by diffusion-weighted imaging, patients with OCD also have increased connectivity between the VS and OFC (Nakamae et al., 2014). This finding is key, because it confirms the presence of aberrant anatomical connectivity between VS and other networks implicated in OCD. Significant negative correlations between NAc volume and YBOCS scores have also been found (Narayanaswamy et al., 2013), and further suggest that these structural abnormalities play a key role in the disorder. In addition to aberrant anatomical connectivity, there is increased resting state functional connectivity between VS and OFC (Harrison et al., 2009; Sakai et al., 2011). It should be noted, however, that one study has shown that VS functional connectivity is not correlated with symptom severity (Sakai et al., 2011), and that some groups have reported decreased connectivity between VS and frontal cortex regions (Admon et al., 2012; Posner et al., 2014).

In addition to changes in connectivity, VS is also associated with aberrant task-evoked activity and altered communication with other brain regions in OCD. Functional imaging studies demonstrate that during anticipation of a monetary reward, healthy controls have increased VS activity. In patients with OCD, however, this activity is significantly reduced (Figee et al., 2011). Decreased VS activity at reward receipt has also been found in patients with OCD (Admon et al., 2012). These findings are critical because they demonstrate dysregulated information processing in the VS of patients, which confirms localization of pathophysiological activity to VS in OCD. These findings also demonstrate abnormal activity related to understanding the consequences of one’s actions, transferring motivational information to action selection, and/or reward processing. Thus, these data support a part of our central thesis: that dysregulation in corticolimbic-VS networks of patients with OCD leads to deficits in processing information critical for selecting behaviors.

### Prediction 2: Aberrant Activity in Ventral Striatal Circuits Causes Compulsive-Like Behaviors

While it has been hypothesized that dysregulated communication in corticolimbic inputs to VS could give rise to compulsive behavior, this is difficult to conclusively prove in humans. Animal studies can be used to address this hypothesis by allowing researchers to investigate causal relationships between abnormal VS inputs and OCD-like phenotypes (Monteiro and Feng, 2015). To perform a direct test of this idea, our group recently modeled mOFC-VS hyperactivity using optogenetics in mice. Brief, repeated, optogenetic stimulation of mOFC terminals in VS progressively led to the development of repetitive grooming behavior that may be relevant to OCD (Ahmari et al., 2013). This repeated stimulation likely led to pathological plasticity, as acute stimulation did not produce this behavioral effect, and the behavioral phenotype emerged in conjunction with an increase in VS firing rates evoked by OFC stimulation. This finding is especially interesting in light of the aforementioned increased functional connectivity of mOFC and VS in patients with OCD, which may be mimicked by the stimulation-induced plasticity. Importantly, both the behavioral and electrophysiological effects of stimulation were reversed by chronic administration of high-dose fluoxetine, a first line treatment for OCD. Together, these data suggest that abnormal functional connectivity in this interregional connection could underlie pathological repetitive behaviors (Ahmari et al., 2013).

Demonstrating causality, not just correlation, between hyperactivity in VS circuitry and OCD-like behaviors in a rodent model strongly suggests that corticolimbic-VS networks could directly contribute to compulsive behaviors in OCD. The spatial specificity of this manipulation is critically important; this optogenetic paradigm selectively stimulated mOFC connections in the VS, so that the behavioral effects of the manipulation originated in a circuit implicated in the pathophysiology of OCD. While these findings demonstrate that a circuit-level manipulation of OFC-VS projections produces OCD-like behaviors in mice (Ahmari et al., 2013), all the corticolimbic inputs detailed in this review could theoretically contribute to compulsive behavioral selection; however, this has not been demonstrated at the present time. While future work should investigate the relationship between dysregulation throughout this network and compulsive-like behaviors, the available data confirm another part of the central thesis of this review: dysregulation of interregional activity in VS circuits is sufficient to cause compulsive-like behavior.

### Prediction 3: Treatments Targeting Ventral Striatal Circuits Ameliorate Affective, Obsessive, and Compulsive Symptoms in Patients with OCD

A final point suggesting that VS circuits contribute to OCD symptomatology is that deep brain stimulation (DBS) in the VS has been shown to improve affective, obsessive, and compulsive symptomatology. Though it should be noted that targeting internal capsule, thalamic peduncle, and subthalamic nucleus has also yielded positive clinical effects (de Koning et al., 2011), we focus here on data from DBS treatment in the VS to convey that this is a locus of effective intervention in OCD.

In an early pilot study, unilateral DBS in the NAc shell improved symptoms in three of four patients tested (Sturm et al., 2003). A subsequent open trial in 10 adult patients demonstrated that bilateral DBS in VS/ventral capsule decreased obsessive-compulsive symptoms from severe to moderate, and improved depression and anxiety ratings three years after treatment was initiated (Greenberg et al., 2006). These studies were highly suggestive that VS DBS could be beneficial for treating OCD. Due to the inevitable difficulties in studying experimental surgical procedures, however, these first studies suffered from low numbers of patients and a lack of double blinding. More recent work has addressed these issues. A combined multi-site, long-term study indicates that VS DBS reduced OCD symptoms in over 60% of the 26 patients who had previously been treatment resistant (Greenberg et al., 2010). Affective symptoms, such as anxiety and depression, were improved in these patients as well (Greenberg et al., 2010). Additionally, Denys et al. (2010) conducted a study with eight months of open treatment, followed by a double-blind crossover phase and resumption of open treatment. Both the open phase and the double-blind crossover phase were associated with decreases in YBOCS scores, anxiety, and depression symptoms. Thus, DBS in the VS of OCD patients has been shown to be an effective treatment for both obsessive-compulsive and affective symptoms.

In addition to improving symptoms, NAc DBS has been shown to reduce functional connectivity with lateral and medial prefrontal cortex (Figee et al., 2013). Thus, DBS can ameliorate not only symptomatology, but also underlying circuit abnormalities. NAc DBS has also been found to decrease D2/3 dopamine receptor binding and dopamine metabolite levels (Figee et al., 2014), and may therefore correct the dopaminergic abnormalities that have been reported in patients with OCD (see above). Additionally, abnormal low frequency (2–5 Hz) oscillations in the frontal cortex are observed in OCD, and NAc DBS reduces the power of these oscillations (Figee et al., 2013). Taken together, these data demonstrate that NAc or VS DBS can reverse neuronal activity disruptions linked to OCD. The reductions in affective, obsessive, and compulsive symptoms, coupled with alterations in neuronal activity, all support the notion that the VS is a locus of effective intervention in OCD.

Insight into the mechanisms underlying this clinical effect can be provided by animal studies. DBS in rat VS subregions receiving preferential innervation from mOFC (dorsal to the anterior commissure) causes increased expression of plasticity markers and neurotrophic growth factors in the mOFC and amygdala (Do-Monte et al., 2013; Rodriguez-Romaguera et al., 2015). It has also been suggested that DBS may override hyperactivity in cortico-striatal projections (Tass et al., 2003); in accord with this hypothesis, NAc DBS decreases orbitofrontal cortex neuronal activity in anesthetized rats (McCracken and Grace, 2007). Taken together, these data suggest that in rodents, DBS in some portions of VS produces electrophysiological changes and plasticity in multiple corticolimbic regions that are linked to compulsivity.

## Closing Statements—The Emerging Role of Corticolimbic-Ventral Striatal Networks in OCD

In closing, we have examined how abnormal activity in amygdala, VTA, and mOFC could lead to aberrant or dysregulated affective representations. These representations may, in turn, impact information processing in the VS, and promote compulsive behavioral selection in patients with OCD. Though areas outside of this network are also clearly important in OCD pathophysiology, our focus on this corticolimbic-VS network complements more extensively studied dorsal striatal circuits by emphasizing the role of affective dysregulation in producing compulsive behavior. In support of this VS model, anatomical or functional abnormalities have been observed in all regions of this network in patients with OCD. Additionally, circuit level disruptions in the VS produce compulsive-like behavior in animal models, and VS DBS can ameliorate both affective and obsessive-compulsive symptomatology in patients with OCD. Much of the data supporting these final two points has emerged within the last decade. Thus, this is a critical period in the study of this severe neuropsychiatric disorder, as new data and treatment approaches contribute to an emerging understanding of the role of corticolimbic-ventral striatal networks in OCD. Collectively, the data highlighted here suggest that the affective dysregulation that is associated with OCD, and aberrant information processing by medial orbitofrontal, VTA, and amygdala inputs to the VS, are critical to producing OCD symptoms. Thus, this network plays a potentially critical role, and is fertile ground for future theoretical and experimental investigations on the origins and treatment of OCD.

## Funding

Funding was provided by the National Institute of Mental Health K08 MH087718, R21 MH096200, R01 MH104255; and by the Brain and Behavior Research Foundation Young Investigator Award, Burroughs Wellcome Fund CAMS Award, MQ Fellows Award, and the McKnight Foundation Scholars Award.

## Conflict of Interest Statement

The authors declare that the research was conducted in the absence of any commercial or financial relationships that could be construed as a potential conflict of interest.
